# Oxidative Stress in Cytokine-Induced Dysfunction of the Pancreatic Beta Cell: Known Knowns and Known Unknowns

**DOI:** 10.3390/metabo10120480

**Published:** 2020-11-24

**Authors:** Anjaneyulu Kowluru

**Affiliations:** Biomedical Research Service, John D. Dingell VA Medical Center, Department of Pharmaceutical Sciences, Eugene Applebaum College of Pharmacy and Health Sciences, Wayne State University, Detroit, MI 48201, USA; akowluru@med.wayne.edu

**Keywords:** proinflammatory cytokines, oxidative stress, NADPH oxidases, Rac1, pancreatic beta cell, diabetes

## Abstract

Compelling evidence from earlier studies suggests that the pancreatic beta cell is inherently weak in its antioxidant defense mechanisms to face the burden of protecting itself against the increased intracellular oxidative stress following exposure to proinflammatory cytokines. Recent evidence implicates novel roles for nicotinamide adenine dinucleotide phosphate (NADPH) oxidases (Noxs) as contributors to the excessive intracellular oxidative stress and damage under metabolic stress conditions. This review highlights the existing evidence on the regulatory roles of at least three forms of Noxs, namely Nox1, Nox2, and Nox4, in the cascade of events leading to islet beta cell dysfunction, specifically under the duress of chronic exposure to cytokines. Potential crosstalk between key signaling pathways (e.g., inducible nitric oxide synthase [iNOS] and Noxs) in the generation and propagation of reactive molecules and metabolites leading to mitochondrial damage and cell apoptosis is discussed. Available data accrued in investigations involving small-molecule inhibitors and antioxidant protein expression methods as tools toward the prevention of cytokine-induced oxidative damage are reviewed. Lastly, current knowledge gaps in this field, and possible avenues for future research are highlighted.

## 1. Introduction

Type-1 diabetes (T1DM) is characterized by an absolute insulin deficiency arising from autoimmune destruction of the pancreatic islet β-cell. It is generally accepted that, during the course of the progression of T1DM, proinflammatory cytokines (e.g., IL-1β, TNF-α, and IFN-γ) are released by infiltrating activated immune cells. However, the exact molecular and cellular mechanisms by which cytokines induce β-cell dysregulation and demise remain only partially understood. In this context, while a host of competing signaling pathways have been proposed to contribute to the beta cell dysfunction under the duress of cytokines, apoptosis is considered as the primary mode of beta cell death in human and mouse models of T1DM [1,2,3]. Interestingly, published evidence also implicates mitochondrial dysfunction as the hallmark of beta cell demise under exposure to proinflammatory cytokines [4,5,6,7,8,9]. The mitochondrial damage via loss of membrane permeability pore transition (MMPT) leads to the release of the mitochondrial cytochrome C into the cytosolic compartment to promote the activation of caspases, culminating in the degradation and functional inactivation of key intracellular proteins that may be necessary for cell proliferation and survival, including G protein prenylating enzymes and nuclear lamins [10,11]. From a mechanistic standpoint, extant studies have suggested potential roles of increased oxidative stress, inflammation, and endoplasmic reticulum (ER) stress as contributors to the islet beta cell dysfunction under the duress of the above-mentioned pathological stimuli. The reader is referred to select reviews highlighting the contributory roles of increased intracellular metabolic stress in the pathology of islet beta-cell destruction under diabetogenic conditions [2,5,12,13,14,15]. 

At the outset, it is important to note that the pancreatic beta cell is relatively more vulnerable to oxidative damage due to the inherent deficiency of a strong antioxidant capacity to counteract the excessive generation of reactive oxygen species (ROS) under conditions of exposure to various pathological insults, including exposure to proinflammatory cytokines. In this context, original contributions from the laboratory of Lenzen and coworkers provided compelling evidence demonstrating poor antioxidant enzymatic machinery in the islet beta cell [16]. Using Northern blot hybridization methodology, these investigators quantified the gene expression of various antioxidant enzymes in mouse tissues. Their data revealed significantly low levels of these genes in pancreatic islets compared to the other tissues studied. For example, Cu-Zn superoxide dismutase and Mn-superoxide dismutase (Mn-SOD) activities in the islet were only 38% and 30%, respectively, of the levels of these enzymes in the liver. Likewise, the expression of the glutathione peroxidase (GPx) gene in the islet was only 15% of the level seen in the liver. Lastly, catalase gene expression was undetectable in the islet. Based on the above findings, these investigators proposed that a relatively low abundance of the antioxidant enzymes in the islet may contribute to its susceptibility to oxidative stress in human and animal diabetes [16]. Several follow-up studies assessed the antioxidant capacity of human islet beta cells. Gurgul-Convey and coworkers demonstrated that the antioxidant enzyme profiles in clonal EndoC-βH1 human beta cells are comparable to those in human and rodent islets. Specifically, these studies have shown relatively high levels of SODs and low levels of H_2_O_2_-inactivating GPx and catalase in these cells [17]. Along these lines, more recent studies by Miki and coworkers [18] demonstrated a significantly high degree of susceptibility of beta cells to oxidative stress compared to alpha cells in the human islet; this is further supported by remarkably low expression levels of GPx and catalase in human beta cells compared to the alpha cells. Together, data accrued in these investigations indicate a clear imbalance between the enzymatic machinery involved in the generation and removal of H_2_O_2_ in pancreatic beta cells in critical intracellular compartments, including mitochondria, leading to cellular dysregulation and demise under the duress of proinflammatory cytokines [17,19,20]. It is well established that IL-1β mediates its cytotoxic effects on rat beta cells via accelerating the NFκB-mediated induction of the inducible nitric oxide synthase (iNOS) and the release of nitric oxide (NO) and downstream signaling events, culminating in cell dysfunction. Interestingly, however, studies in clonal EndoC-βH1 human beta cells, human islets, and mouse islets (not rat islets) implicate NO-independent effects of cytokines in the induction of metabolic stress and cellular dysregulation [17,21,22,23]. These observations clearly provide additional support and clarification for the differences reported in earlier studies with regard to differential responses and effects of proinflammatory cytokines on rat, mouse, and human islets and a wide variety of clonal beta cell lines [21]. Based on the above discussion, a clear picture is emerging to suggest critical roles for an inefficient handling of high levels of intracellularly generated H_2_O_2_ in the beta cell under the duress of cytokines, leading to the pathology of islet beta cell dysfunction and demise. Altogether, a host of intracellularly generated reactive oxygen species (ROS, e.g., superoxide radicals, hydroxyl radicals, and H_2_O_2_) and reactive nitrogen species (RNS, e.g., NO and peroxynitrite) could play significant roles in the induction of proinflammatory cytokine-induced damage to the islet beta cell [20]. Lastly, superoxide radicals, which are generated by NADPH oxidases (Noxs), undergo dismutation by SODs to promote generation of H_2_O_2_ in relevant subcellular compartments (e.g., cytosol) in the cytokine-challenged beta cell, leading to damage and loss of functional beta cell mass (see below). The reader is referred to previously published articles for additional details highlighting the deficiency of a robust antioxidant status and the activation of NADPH oxidases as the potential contributing factors for islet beta cell failure and demise under the duress of diabetogenic conditions [20,24,25,26]. 

## 2. NADPH Oxidases Play Key Roles in Islet Function in Health and under Stress Induced by Cytokines

**Figure 1:**
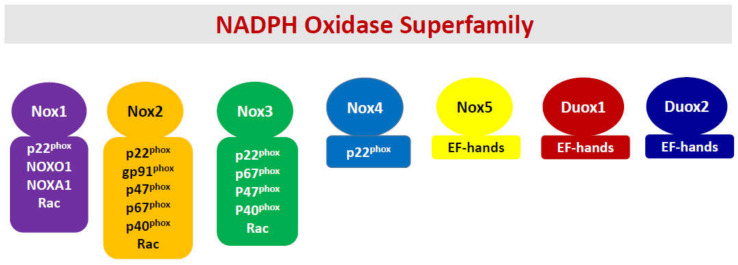
A schematic representation of various members and their subunit composition of the Nox superfamily.

In the back drop of the above discussion about the unique situation the islet beta cell faces, namely poor antioxidant defense, and a high degree of intracellular oxidative stress, which is created under diabetogenic conditions, I have overviewed our current understanding of the roles of NADPH oxidases (Noxs) in the onset of metabolic dysfunction of the beta cell under the duress of exposure to pro-inflammatory cytokines. The Nox superfamily represents a class of flavocytochromes that promote transport electrons through biological membranes and catalyze the cytosolic NADPH-dependent reduction of molecular oxygen to superoxide radicals [25,26,27,28,29,30,31,32,33,34,35]. Interestingly, however, despite a considerable degree of similarity in their ability to generate high levels of superoxide radicals under metabolic stress conditions, they significantly differ in structural composition, subcellular distribution, and response to specific external stimuli (Figure 1). Briefly, the Nox superfamily consists of seven members, namely Nox1-5, dual oxidases 1 (Duox1), and 2 (Duox2). Nox1-3 are membrane bound and require other cytosolic core proteins for holoenzyme assembly and activation. For example, the regulatory components for Nox1 include p22^phox^, NOX organizers NOXO1 and NOXA2, and the small G protein Rac1. The Nox2 holoenzyme is comprised of membranous cytochrome b558, a heterodimer consisting of p22^phox^, gp91^phox^, and the cytosolic core of proteins, including p40^phox^, p47^phox^, p67^phox^, and small G protein Rac1. It has also been proposed that Rap1, a membrane-associated small G protein, contributes to functional regulation of Nox2 holoenzyme [36]. However, potential regulatory roles of Rap1 in functional activation of Nox2 or other forms of Nox in the pancreatic beta cell remain to be elucidated further. 

The Nox3 is comprised of p22^phox^, NOXO1, NOXA1, and Rac1. Interestingly, Nox4, which is localized intracellularly, requires only p22^phox^ but no cytosolic core of proteins. From a mechanistic standpoint, its activity is regulated by its expression, and hence, it is considered constitutively active [29]. It is important to note that Nox 1-4 have a critical requirement for p22^phox^ and Nox1-3 require Rac1 for optimal catalytic function [33]. Lastly, Nox5, Duox1, and Duox2 are associated with the plasma membrane and do not require the intermediacy of cytosolic core of proteins [29,33]. However, these Noxs have calcium-binding motifs (EF hands) for optimal activation (Figure 1). Lastly, it is noteworthy that the Nox5 gene is not expressed in mice and rats [32]. The reader is referred to recent reviews that highlight subunit composition, regulation of individual subunit function via post-translational modifications, translocation to the membrane, holoenzyme assembly, and functional activation of individual members of the Nox superfamily [25,26,27,28,29,30,31,32,33,34]. It may be germane to point out that investigations from multiple laboratories have reported expression of Nox1 [37,38,39], Nox2 [26,40,41,42,43], Nox4 [41,44], and Nox5 [45] in human islets, thus making these oxidases potential regulators of islet function and dysfunction in health and disease. Lastly, it should be noted that, of the seven members that belong to the Nox superfamily, only Nox1 and Nox2 are studied extensively in the context of their roles in cytokine-mediated dysregulation of the islet beta cell (see below). Therefore, this review will focus on potential roles of Nox1 and Nox2 in the onset of metabolic defects in the pancreatic islet beta cell under the duress of proinflammatory cytokines.

## 3. Regulatory Roles of Noxs in Physiological Insulin Secretion

Published evidence from several laboratories has provided convincing evidence on the expression of various members of the Nox superfamily, specifically Nox1, Nox2, and Nox4, in pancreatic beta cells. Examples of relevant studies are highlighted here. Using a variety of complementary experimental approaches, Rebeleto and coworkers [40] reported expression of various subunits of Nox2 in clonal pancreatic beta cells, and rodent and human islets. In addition, they demonstrated inhibition of glucose metabolism and GSIS in these cells following inhibition of Nox2, thus suggesting regulatory roles of Nox2 in physiological insulin secretion. Along these lines, studies by Uchizono and Sumimoto [46] showed an association of Nox1, Nox2, Nox4, and p22^phox^ with the membrane in rodent pancreatic islets. Furthermore, they reported cytosolic distribution of p47^phox^, Noxo1 (homologue of p47^phox^), Noxa1 (homologue of p67^phox^), and p40^phox^ in these cells. Compatible with findings of Rebeleto and coworkers [40], these studies have also observed inhibition of GSIS by diphenyleneiodonium (DPI), a known inhibitor of Nox2. In another set of investigations, Oliviera and coworkers [47] studied subunit expression and functional regulatory roles of Nox2 in islet beta cell function. Using RT-PCR and/or Western blotting methods, they reported expression of various Nox2 subunits in rat pancreatic islets. Using immunohistochemical approaches, they demonstrated glucose-induced Nox2 activity in these cellular preparations. Lastly, mechanistic studies involving phorbol myristate acetate, a known activator of protein kinase C (PKC), and GF109203X, a specific inhibitor of PKC, further implicated novel roles of PKC in glucose-mediated regulation of Nox2 in pancreatic islets [47]. Together, data from the above studies have suggested acute regulatory roles for the Nox subfamily of enzymes in physiological insulin secretion. The following sections will highlight our current understanding of regulatory roles of various members of the Nox family in cytokine-mediated dysregulation of the islet beta cell, which is the primary focus of this review.

## 4. Regulatory Roles of Nox1 in Cytokine-Induced Dysfunction of the Beta Cell

In a series of investigations, Weaver and coworkers addressed potential roles of Nox1 in cytokine-induced islet beta-cell dysfunction. In a study published in 2012, they reported a significant increase in the expression of Nox1 in human islets, mouse islets, and clonal beta cell lines exposed to a mixture of proinflammatory cytokines [37]. A significant increase in the expression of monocyte chemoattractant protein-1 (MCP-1), ROS generation, loss in GSIS, and associated increase in cell death was also reported under these conditions. Coprovision of pharmacological inhibitors of Nox (apocynin, DPI, and a dual selective inhibitor of Nox1/Nox4) markedly attenuated cytokine-induced MCP-1 expression and ROS generation. Follow-up studies by these investigators provided additional support for regulatory roles of Nox1 in cytokine-induced dysregulation of the islet beta cell [38]. They revealed that exposure of murine beta cells to ML171, a selective inhibitor of Nox1, significantly impeded cytokine-induced ROS generation, caspase-3 activation, and cell death via apoptosis. Moreover, ML171 significantly prevented loss in GSIS induced by proinflammatory cytokines in both clonal beta cells and isolated mouse islets. Based on these data, these researchers concluded that cytokine-induced metabolic dysfunction of the islet beta cell involves Nox-1-mediated increase in ROS generation and associated intracellular oxidative stress. They also proposed that targeting of Nox-1 might serve as a valuable approach to protect proinflammatory cytokine-induced metabolic dysfunction of the beta cell in diabetes [38]. Subsequent investigations by these researchers have further affirmed the contributory roles of Nox-1 in intracellular generation of ROS and oxidative stress in pancreatic beta cells [48]. Forced expression of Nox1 in pancreatic beta cells resulted in increased generation of ROS, loss in GSIS, and increased cell death via apoptosis. It is important to note that these cellular events are comparable to those observed in beta cells under the duress of exposure to proinflammatory cytokines since shRNA-mediated suppression of Nox-1 markedly prevented deleterious effects of cytokines. Taken together, these studies have identified Nox-1 as a potential therapeutic target in the prevention of cytokine-induced islet dysfunction in diabetes. 

Published evidence suggests that the 12-lipoxygenase (12-LO), which catalyzes the oxidation of fatty acids to their respective hydro peroxides, plays novel roles in cytokine-mediated dysregulation of the pancreatic islet beta cell [49,50]. In this context, studies by Weaver and coworkers demonstrated that 12-hydroxyeicosatetranoic acid (12-HETE), a product of 12-LO, significantly increased the expression of Nox-1 in human islets [37]. More importantly, NCTT-956, a selective inhibitor of 12-LO, but not its inactive analog (NCTT-695), markedly suppressed cytokine-induced Nox-1 expression, ROS generation, and caspase-3 activation in clonal beta cell preparations. Taken together, these findings provide a novel model, which implicates key regulatory roles of the 12-LO-Nox1 signaling axis in proinflammatory cytokine-induced metabolic dysregulation of the islet beta cell. 

## 5. Regulatory Roles of Nox2 in Cytokine-Induced Dysfunction of the Beta Cell

As stated above, considerable efforts were made previously to assess the regulatory roles of phagocyte-like NADPH oxidase (Nox2) in cytokine-induced metabolic dysregulation of the islet beta cell [25,26]. Briefly, Nox2 has been shown to play key roles in phagocytosis by professional phagocytic cells (e.g., neutrophils, eosinophils, monocytes, and macrophages). The Nox2 holoenzyme is a highly regulated membrane-associated protein complex, the activation of which results in the generation of large quantities of intracellular ROS, which, in turn, promote activation of several downstream signaling events, including mitochondrial dysfunction, culminating in cell demise. Along these lines, several mechanisms have been put forth for the generation of ROS and associated oxidative stress in a variety of non-phagocytic cell types, including the islet β-cell [25,26]. As depicted in Figure 1, the Nox2 is a multicomponent system, which is comprised of membranous and cytosolic cores. The membrane-associated catalytic core is comprised of gp91^phox^ and p22^phox^. The cytosolic core of Nox2 (also referred to as the regulatory core) is comprised of p40phox, p47^phox^, p67^phox^, and Rac. Following cell stimulation, the cytosolic (regulatory) core proteins translocate to the membranous compartment to associate with the catalytic core for the formation of Nox2 holoenzyme, resulting in the catalytic activation of Nox2 and generation of ROS. Several mechanisms, including phosphorylation of p40^phox^, p47^phox^, and p67^phox^, have been proposed as requisite steps for the translocation of the cytosolic core to the membrane. In the case of Rac1, it appears that its activation (GTP-bound conformation), mediated by specific guanine nucleotide exchange factors (GEFs, e.g., Tiam1), favors its association with p67^phox^, thus enabling the translocation of Rac1-p67^phox^ dimer to the membrane [11,26,51]. The experimental findings described in the following section will highlight potential contributory roles of Nox2 in the cascade of events leading to proinflammatory cytokine-induced metabolic dysfunction of the islet beta cells. 

Using primary mouse islets and insulin-secreting BRIN-BD11 β-cells, Michalska and Newsholme reported significant inhibition of GSIS and an increase in the expression of p47^phox^ and iNOS in these cells following exposure to proinflammatory cytokines [52]. Interestingly, coprovision of antioxidants, such as SOD, catalase, and N-acetylcysteine (NAC), markedly suppressed effects of H_2_O_2_ or palmitate but not those elicited by proinflammatory cytokines. These studies also demonstrated a significant prevention of deleterious effects of cytokines and H_2_O_2_ following pharmacological inhibition of Nox and/or iNOS. It was concluded that H_2_O_2_ might play contributory roles in positive feedback redox sensitive regulation of β-cell dysfunction via its effects on Nox and iNOS [52]. 

Subasinghe and coworkers examined the roles of Nox2 in cytokine-induced metabolic dysfunction of the islet beta cell [53]. Specifically, they investigated the contributory roles of Rac1 in the onset of beta cell dysfunction under the duress of cytokines. They observed a significant increase in Nox2 activation, ROS generation, and in the expression of p47^phox^ subunit, but not p67^phox^ subunit, in INS-1 832/13 cells following exposure to a mixture of proinflammatory cytokines. The hypothesis that Nox2 is involved in cytokine-induced Nox2 activation and ROS generation was further confirmed using siRNA-p47^phox^. These observations were further validated by using pharmacological inhibition of Nox2 using apocynin. Specific inhibitors of Rac1, namely NSC23766 (inhibitor of Tiam1-Rac1 signaling) and GGTI-2147 (inhibitor of prenylation of Rac1), significantly suppressed the cytokine-induced Rac1 activation and alterations in MMPT in these cells. Data accrued from these studies also suggested that the cytokine-mediated Tiam1/Rac1 signaling pathway may not be necessary for iNOS expression and NO release since NSC23766 failed to exert any significant effects on IL-1β (or a mixture of cytokines)-induced NO release in INS 832/13 cells [53]. Collectively, these findings suggested novel roles for the Tiam1-Rac1 axis in cytokine-induced ROS but not NO generation in beta cells under the duress of cytokines. In addition, these studies have provided the first evidence to suggest that prenylation of Rac1 is a requisite for cytokine-mediated effects. Based on these findings it was suggested that the combined effects of intracellularly generated NO (via activation of iNOS) and ROS (via activation of Nox2) could contribute to alterations in mitochondrial function, leading to caspase-3 activation and metabolic dysfunction of the β-cell [53]. 

Along these lines, Mohammed and coworkers [54] demonstrated a time-dependent phosphorylation of p47^phox^ in INS-1 832/13 cells exposed to a mixture of cytokines. A significant increase in the expression of gp91^phox^ was also noted under these conditions. Lastly, 2-Bromopalmitate, a known inhibitor of protein palmitoylation, markedly attenuated cytokine-induced, Nox2-mediated ROS generation, and iNOS-mediated NO generation [54]. Together, these studies identified palmitoyltransferase as a target for inhibition of cytomix-induced oxidative and nitrosative stress in the pancreatic beta cell. Based on the NSC23766-mediated inhibition of Rac1, it is likely that cytokine-induced activation of iNOS and Nox2 are under the control of (at least) two G proteins that require palmitoylation [53,54] (see below). 

Previously published evidence suggests that phosphorylation of p47^phox^ may be mediated by PKC, a signaling event that has been implicated in the translocation of this subunit to the membranous core for Nox2 holoenzyme assembly [33,55,56,57]. In further support of this hypothesis, studies by Morgan et al. demonstrated partial restoration of IL-1β-induced ROS to normal levels following exposure to GF109203X, a known inhibitor of PKC [58]. These findings support the formulation for a multifactorial regulation of Nox2 subunits by proinflammatory cytokines, leading to its activation and ROS generation, and culminating in the activation of downstream signaling events involved in cell dysfunction. 

Lastly, earlier studies in animal models of impaired insulin secretion and diabetes affirmed critical regulatory roles of Nox2 in cytokine-induced metabolic dysregulation of the islet. For example, Xiang and coworkers demonstrated that deficiency of Nox2 decreases beta cell destruction and preserves islet function in STZ-induced diabetes by reducing ROS production, immune response, and β-cell apoptosis [59]. Studies of Veluthakal and coworkers demonstrated that administration of NSC23766, a known inhibitor of the Tiam1-Rac1-Nox2 signaling pathway, significantly prevented the development of spontaneous diabetes in the non-obese diabetic (NOD) mice [60]. In addition, they observed that NSC23766 treatment markedly suppressed Rac1 expression, activity, and ER stress in NOD islets. Based on the findings, it was concluded that the Tiam1-Rac1-Nox2 signaling pathway plays critical regulatory roles in the onset of spontaneous diabetes in the NOD mouse model. Collectively, findings from both in vitro and in vivo provide compelling evidence for critical regulatory roles of Nox2 in proinflammatory cytokine-induced ROS generation and metabolic dysfunction culminating in the onset of islet dysfunction and diabetes.

## 6. Roles of Nox3, Nox4, and Nox5 in Cytokine-Induced Dysfunction of the Beta Cell

It is noteworthy that a recent literature search (Pubmed; October 2020) indicated no clear evidence of contributory roles of Nox3 in islet function. With respect to regulatory roles of Nox4, Wang et al. undertook a pharmacological approach to assess the roles of various Noxs (Nox-1, Nox-2, and Nox-4) in human pancreatic beta-cell dysfunction induced under a variety of diabetogenic conditions, including exposure to proinflammatory cytokines [41]. They demonstrated that pharmacological inhibition of Nox (using DPI, dapsone, GLX351322, and GLX481372) attenuated ROS levels, caspase activation, and loss in cell viability in human islets under the duress of glucolipotoxic conditions. ML171, a specific inhibitor of Nox1, failed to exert any significant effects on cellular dysfunction induced by diabetogenic condition, including exposure to cytokines in human islet cells. Furthermore, Phox-I2, a known inhibitor of Nox2, elicited partial protective effects induced by glucolipotoxic conditions in human islets without significantly affecting cytokine-induced dysfunction of the islet beta cell. Lastly, GLX7013114, a highly selective inhibitor of Nox4, exhibited protective effects in human beta cells under the duress of glucolipotoxicity and cytokine exposure. Based on these findings, the authors proposed that Nox4 mediates pro-apoptotic effects in intact islets under stressful conditions and that selective Nox4-inhibition may be a therapeutic strategy in type 2 diabetes [41]. Interestingly, the findings that Nox1 and Nox2 are not involved in cytokine-induced effects are in contrast to findings of the studies described in the above sections. Additional investigations are needed to explain the differences between these experimental outcomes and conclusions drawn in these studies.

It is noteworthy that, in a manner akin to Nox3, a recent review of the literature yielded very limited details on the potential regulatory roles of Nox5 in cytokine-induced dysregulation of the islet beta cell. Interestingly, however, Nox5 does appear to contribute to islet beta cell dysfunction under the duress of other pathological stimuli. For example, Bouzarki and coworkers [45] reported expression of Nox5 in somatostatin-containing delta cells in human islets under basal conditions. Selective depletion of expression of Nox5 using siRNA-Nox5 significantly attenuated GSIS, suggesting novel regulatory roles of Nox5 in physiological insulin secretion. Furthermore, long-term exposure of human islets to high glucose resulted in increased expression of Nox5 in the beta cells. Lastly, the degree of impairment in GSIS following high-fat feeding was markedly aggravated in animals in which Nox5 was conditionally increased in beta cells. Taken together, these investigations provided novel insights into the roles of Nox5 in promoting crosstalk between various cell types (i.e., the paracrine relationship between delta and beta cells) of the pancreatic islet under physiological conditions. Their findings also implicated novel roles for Nox5 in promoting vulnerability of the islet beta cell for damage under various pathological conditions [45]. As stated above, potential regulatory roles of Nox5 in proinflammatory cytokine-induced metabolic dysfunction of the islet beta cell remain to be studied further. It should also be kept in mind that Nox5 is not expressed in rats and mice [27,32]. Therefore, future studies should be focused on human islet cells in which Nox5 appears to be expressed and regulated under defined experimental conditions.

Taken together, it is evident that Nox1, Nox2, and Nox4 play critical regulatory roles in cytokine-induced metabolic dysregulation and demise of the islet beta cell. Additional investigations are needed to further explore the regulatory roles of other Nox forms, namely Nox3 and Nox5, in the cascade of events leading to islet dysfunction under the duress of cytokines. More importantly, studies in human islet cells are needed to ascertain the translational significance of these signaling pathways in the onset of beta cell dysfunction in human diabetes. 

**Figure 2:**
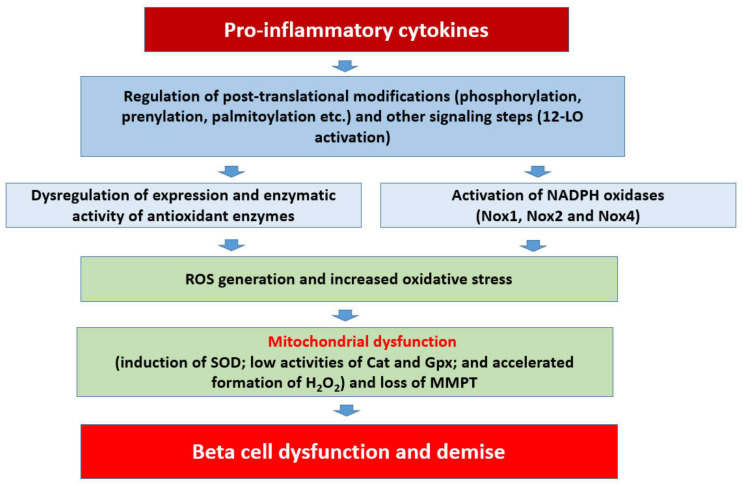
Potential signaling mechanisms involved in cytokine-induced Nox-mediated dysregulation of the islet beta-cell.

Based on the available evidence on regulatory roles of Noxs, a working model is proposed (Figure 2), which states that chronic exposure of pancreatic islet beta cells to proinflammatory cytokines results in the activation of at least three members of the Nox superfamily (Nox1, Nox2, and Nox4), leading to the generation of ROS and the onset of intracellular oxidative stress. Several lines of evidence (highlighted above) suggest that functional regulation of specific subunits of Nox is precisely mediated via post-translational modifications, including phosphorylation (e.g., p47^phox^, p60^phox^ etc.), prenylation, and palmitoylation (e.g., Rac1). Such modifications are a requisite for their translocation to the membrane for association with the membranous core of Nox to complete holoenzyme assembly and functional activation of the Nox. As stated in the above sections, the 12-LO pathway also plays a critical regulatory role in the initiation of metabolic signals and events culminating in islet beta cell dysregulation following exposure to proinflammatory cytokines. It should be noted that Rac1 is an integral part of Nox1 and Nox2 but not Nox4 (Figure 1). Therefore, Rac1-independent mechanisms must underlie cytokine-induced regulation of Nox4. It is proposed that sustained intracellular oxidative stress and an imbalance in the ROS scavenging steps lead to mitochondrial dysfunction and activation of proapoptotic caspases (e.g., caspase-3), culminating in the cleavage and inactivation of pro-survival proteins, and resulting in accelerated beta cell dysfunction and demise [10,11].

## 7. Potential Crosstalk between iNOS and Nox2 Signaling Pathways in the Onset of Cytokine-Induced Metabolic Dysregulation of the Islet Beta Cell

Peroxynitrite (PN) is generated rapidly in the cell from the interaction between NO and superoxide radicals. Increased intracellular PN levels leads to accelerated oxidation of proteins, lipids, DNA, as well as damage to intracellular organelles, including the mitochondria. Indeed, such cellular events are involved in a variety of pathological states, including cardiovascular, neuronal, and metabolic diseases [61,62,63,64]. Under healthy conditions, PN is short-lived and considered not harmful. However, under pathological conditions, a high degree of production of iNOS-derived NO and Nox-derived ROS and superoxide radicals could lead to substantially high levels of PN. As recently reviewed by Pacher and coworkers [61], a modest increase in superoxide radicals and NO by 10-fold results in a 100-fold increase in PN. The authors suggested that under proinflammatory conditions, the generation of NO and superoxide is expected to increase by 1000-fold, which results in remarkably high levels (1,000,000-fold) of PN. Indeed, such an insult would be much more damaging to the islet beta cell, which is inherently ill equipped with adequate antioxidant defense mechanisms [16,20,24,65].

Considerable debate still exists concerning potential contributory roles of PN in cytokine-induced dysfunction of the pancreatic islet beta cell. From a historical perspective of this topic, original investigations by Delaney and coworkers provided the first evidence to suggest sensitivity of human islets to PN leading to dysfunction and demise [66]. They reported that acute exposure of human islets to PN leads to inhibition of glucose oxidation and accelerated DNA damage (strand breaking). Significant alterations in cell ultrastructure, including organelle degradation, mitochondrial swelling, and matrix loss, were also noted under these conditions. Cell death analysis studies suggested necrotic, rather than apoptotic, demise of these cells following exposure to PN. Data from studies of Lakey et al. provided evidence for critical regulatory roles of PN in cytokine-mediated dysfunction of human pancreatic islets [67]. They reported high levels of nitrotyrosine, a marker of PN, in islet cells exposed to a mixture of proinflammatory cytokines. In a manner akin to the effects of cytokines, provision of exogenous PN led to nitrotyrosine formation and cell dysfunction. Lastly, co-provision of guanidinoethyldisulphide (GED), a known inhibitor of iNOS and scavenger of PN, attenuated cytokine-induced NO release, H_2_O_2_ production, nitrotyrosine formation, and associated cell dysfunction. Based on these observations, it was concluded that PN formation is causal to cytokine-mediated effects on human islets. Subsequent studies by Suarez-Pinzon et al. demonstrated that GED significantly reduced the onset of diabetes in the NOD mouse model [68]. Furthermore, GED markedly suppressed NO and nitrotyrosine formation and cell demise in NOD mouse islets incubated with proinflammatory cytokines. It was concluded that increases in intracellular superoxide radicals and NO levels culminate in the formation of PN, which, in turn, leads to beta cell destruction in autoimmune diabetes. Further investigations by Mabley and coworkers [69] affirmed the therapeutic efficacy of GED in preventing the onset of islet dysfunction in these experimental models. Contrary to the evidence described above, more recent findings from Corbett’s laboratory have suggested that NO, but not PN, mediates cytokine-induced dysfunction of the islet. Evidence is also presented by these researchers that endogenous cytosolic peroxiredoxin 1 (Prdx1) affords protection to the beta cell against intracellularly generated ROS and reactive nitrogen species (RNS) [70]. Collectively, it is evident from the above narrative that cytokine-induced NO and ROS levels exert deleterious effects singly, or in combination, on beta cell function. As stated above, it is important to note that data accrued from investigations involving human islets revealed that the human beta cells are not equipped to generate NO as they do not express iNOS. However, they have been shown to elicit sensitivity to RNS, when co-provided exogenously, resulting in increased interaction with super oxide radicals, intracellularly culminating in beta cell dysfunction. [17,21,22,23]. These aspects must be kept in mind in the interpretation of experimental data from earlier investigations, and in planning future studies to further decipher roles of this signaling module and PN in the cascade of events leading to cytokine-induced damage to the islet beta cell.

## 8. Restoration of Intracellular Redox Environment Prevents Cytokine-Induced Metabolic Defects in the Beta Cell 

Despite the rapid advances in the field, several knowledge gaps still exist, specifically in addressing the fact that the beta cell antioxidant capacity and its ability to scavenge H_2_O_2_ are relatively low compared to other cell types. Extant studies have utilized many approaches, including provision of antioxidants to “rescue” the beta cell against noxious effects of diabetogenic stimuli, including proinflammatory cytokines. For example, using insulin-secreting BRIN-BD11 beta cells, Michalska et al. reported beneficial effects of antioxidants, such as SOD, catalase, and NAC, against deleterious effects of H_2_O_2_ but not cytokines [52]. In a series of methodical investigations Tran et al. demonstrated that adenoviral overexpression of glutamylcysteine ligase, an enzyme involved in the de novo biosynthesis of glutathione, protects pancreatic islets against IL-1β-induced loss in GSIS; such effects were shown to be via an increase in intracellular reduced glutathione (GSH) levels [71]. Studies by Gurgul and coworkers provided evidence for significant protection of insulin-secreting RINm5F cells overexpressing mitochondrial catalase against proinflammatory cytokine-induced cell death; these findings affirm contributory roles for mitochondrial ROS in cytokine-mediated effects [72]. Using stable expression and suppression of MnSOD in RINm5F cells, Lortz and associates further validated the hypothesis that an imbalance between superoxide generation and H_2_O_2_ detoxification enzymes dictates the vulnerability of beta cells to cytokine-induced damage [73]. Overexpression of catalase in the mitochondria has also been shown to afford protection against cytokine-induced nitro-oxidative stress and demise in insulin-producing RINm5F cells [19]. Interestingly, expression of an endoplasmic reticulum-targeted and luminal-active catalase variant (ER-catalase N244) provided protection in INS-1E cells against H_2_O_2_- but not cytokine-induced toxicity [74]. Lastly, studies of Mehmeti and coworkers [75] revealed that overexpression of mitochondrial-specific catalase (MitoCatalase) prevented cytokine-induced alterations in Bax/Bcl-2, and the downstream signaling events, including cytochrome C release and activation of executioner caspases 3 and 9. Indeed, data from the above investigations provide support for the overall hypothesis that deficiencies and/or alterations in ROS scavenging mechanisms within the mitochondrial compartment increase the susceptibility of the islet beta cell to cytokine-induced damage.

Several other candidate genes were examined for their protective effects of beta cells against cytokine insult. For example, Stancill and coworkers recently reported that pharmacological inhibition of Prdx1 (by conoidin A) or siRNA-mediated depletion of expression of endogenous Prdx1 significantly increased the vulnerability of clonal beta cells and rat islets to H_2_O_2_ and PN. Based on these findings, the authors concluded that Prdx1 provides defense against intracellularly generated nitroso and oxidative stress in the cytokine-challenged beta cell [70]. In this context, Wolf and coworkers investigated the potential cytoprotective roles of mitochondrial Prix III, a thioredoxin-dependent peroxide reductase, against oxidative and nitrosative stress in rat insulinoma cells either over- or under-expressing Prdx III, under the duress of a variety of stimuli, including H_2_O_2_ and proinflammatory cytokines [76]. Their findings revealed a significant resistance in Prdx III-expressing cells to these stimuli as evidenced by a marked suppression of iNOS gene expression and the downstream signaling events, including caspase-9 and caspase-3 activation. Together, the above investigations revealed key protective roles of the Prdx family of proteins against cytokine-mediated dysregulation of the islet beta cell. Lastly, using knockout animal models, studies from Lammert’s laboratory demonstrated that antioxidant protein DJ-1, which is encoded by the Parkinson’s disease gene PARK7, affords protection of beta cells against proinflammatory cytokine-mediated cell dysfunction and demise [77,78]. Altogether, thee examples of studies cited above affirm support for the proposal that maintenance (or boosting) of intracellular antioxidant capacity (defense mechanisms) of the beta cell represents a viable therapeutic option to protect the beta cell against cytokine insult.

## 9. Conclusions

A growing body of evidence, in in vitro and in vivo model systems, implicates intracellularly generated oxidative stress as one of the contributing factors in the cascade of events leading to cytokine-induced metabolic dysfunction and demise of the islet beta cell. In this review, I attempted to summarize the known evidence in support of regulatory roles of Noxs, specifically Nox1 and Nox2, in cytokine-induced alterations in the metabolic functions of the islet. Evidence in favor of a regulatory role for Nox4 is emerging, but potential roles of Nox3 and Nox5 in this signaling cascade remain relatively poorly understood. Several pharmacological approaches have been employed to decipher the roles of Noxs, iNOS, and 12-LO as mediators of cytokine-induced metabolic dysregulation of the islet beta cell (Table 1). Indeed, such approaches have provided useful insights into these pathways as potential targets to prevent/halt the metabolic defects. Potential regulatory roles of PN as a mediator of beta cell damage under the duress of cytokines remains to be examined further. In addition, much is unknown with regard to signaling mechanisms and regulatory proteins/factors that promote crosstalk between iNOS-12-LO-Nox2 signaling pathways in the cascade of events leading to cytokine dysregulation of the islet beta cell. These aspects need to be addressed further. 

**Figure 3:**
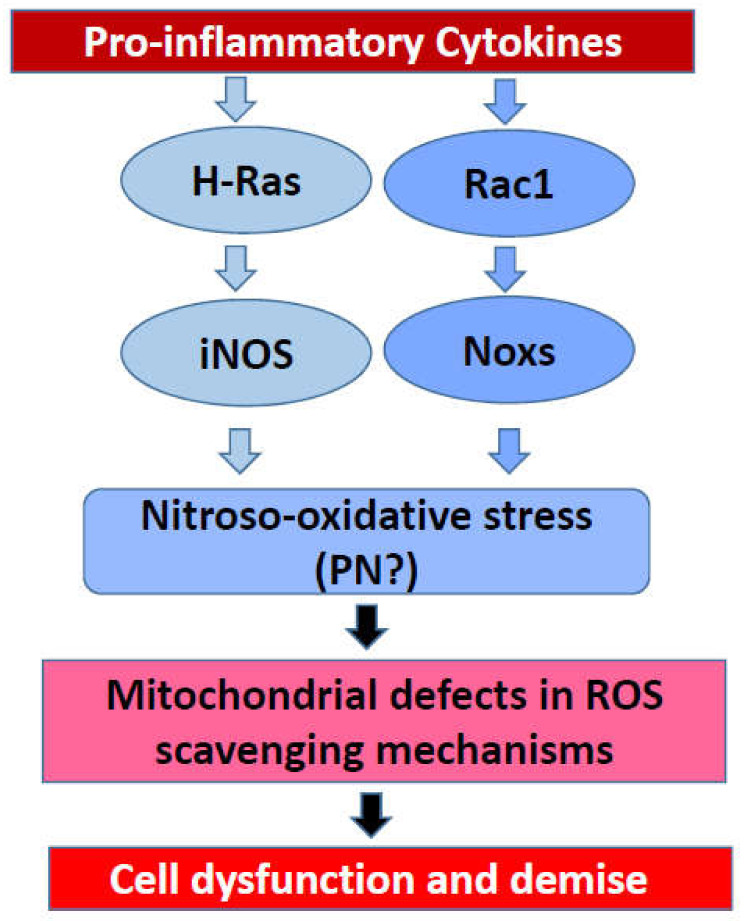
Potential involvement of small G proteins in cytokine-induced NO release and ROS formation leading to mitochondrial dysfunction and demise of the rat islet beta cell.

Based on the evidence, principally accrued in our laboratory on the regulatory roles of small G proteins in the generation of iNOS-derived ROS and Nox2-derived ROS, I propose a model for a potential crosstalk between these signaling pathways in eliciting damage to the rat islet beta cell following exposure to proinflammatory cytokines (Figure 3). Briefly, using pharmacological approaches, it was demonstrated that IL-1β-induced iNOS expression and NO release are under the control of H-Ras, a small G protein belonging to the Ras superfamily of G proteins [80,81]. These findings were further confirmed with bacterial toxins that promote glucosylation and inactivation of small G proteins [80,81]. Interestingly, data accrued from the investigations involving bacterial toxins suggested that activation of Rho G proteins (e.g., Rac1) is not a requisite for IL-1β-induced iNOS expression and NO acid release [81]. Subsequent studies involving specific inhibitors of Rac1 (e.g., NSC23766) further affirmed the postulation that Rac1 is involved in ROS generation but not NO production in a cytokine-challenged beta cell [53]. The combined effects of intracellularly generated NO (due to activation of iNOS) and ROS (due activation of Nox) could contribute to maximal damage of the mitochondrial membrane properties leading to metabolic dysfunction of the beta cell (Figure 2 and Figure 3). It is emphasized that this model, involving H-Ras, may not be applicable to the human beta cells since they do not express the iNOS-NO signaling pathway, and yet exert sensitivity to NO, which is secreted from activated immune cells. The overall concept of intracellular generation of PN and its effects on mitochondrial dysregulation, loss in GSIS, and apoptotic demise of the islet beta cell under the duress of cytokines was proposed earlier [85]. Additional studies are needed to further substantiate this formulation. It is my hope that the future advances in the field of Nox biology will help not only in our current understanding of this class of enzymes but also in the development of small-molecule compounds (via combinatorial chemistry approaches) with a high degree of specificity to inhibit the iNOS-Nox-LO pathways. The development of methodologies to boost the overall antioxidant capacity of the islet beta cell is also warranted. Lastly, novel approaches to accelerate detoxification of intracellularly generated H_2_O_2_ in specific subcellular compartments (e.g., cytosol and mitochondria) might prove valuable in preventing cytokine-mediated dysfunction and demise of the pancreatic islet beta cell.

## Figures and Tables

**Table 1:** Inhibitors of the Nox, iNOS, and 12-LO signaling pathways employed in studies highlighted in this review.

Inhibitor	Mechanism(s) of Action	Reference
GGTI-2147	Inhibitor of prenylation of G proteins (Rac1)	[53]
NSC23766	Inhibitor of Tiam1-Rac1 signaling pathway	[53]
Manumycin	Pan inhibitor of farnesylation of G proteins (Ras)	[79,80,81]
Damnacanthal	Pan inhibitor of farnesylation of G proteins (Ras)	[80,81]
Cerulenin	Inhibitor of palmitoylation of G proteins	[79]
2-bromopalmitate	Inhibitor of palmitoylation of G proteins	[54,79]
DPI	Pan inhibitor of Noxs	[37,41,58]
Apocynin	Pan inhibitor of Noxs2	[37,53,54]
Gp91ds-tat	Inhibitor of Nox2	[82]
Guanidinoethylsulphide	Inhibitor of iNOS and scavenger of PN	[67,68]
GLX7013114	Specific inhibitor of Nox4	[41]
GLX351322	Selective inhibitor of Nox4 (inhibits other Noxs)	[41]
GLX481372	Selective inhibitor of Nox4 (inhibits other Noxs)	[41]
Dapsone	Inhibits expression/activity of Nox4 and DUOX1	[41]
ML171	Inhibitor of Nox1	[38,41]
Phox-I2	Inhibitor of Nox2	[41]
ML351	Inhibitor of 12/15-LO	[83]
NCTT-956	Inhibitor of 12-LO	[37,84]
GF109203X	Inhibitor of PKC	[58]

## Abbreviations

DPI	diphenyleneiodonium
Duox1	dual oxidase1
Duox2	dual oxidase2
EF hands	calcium binding domains or motifs
ER stress	endoplasmic reticulum stress
FTase	farnesyltransferase
GEFs	guanine nucleotide exchange factors
GSH	reduced glutathione
GSIS	glucose-stimulated insulin secretion
12-HETE	12-hydroxyeicosatetranoic acid
IFNγ	interferon γ
IL-1β	interleukin 1β
iNOS	inducible nitric oxide synthase
12-LO	12-lipoxygenase
MCP-1	monocyte chemoattractant protein-1
MMPT	mitochondrial membrane pore transition
Nrf2	nuclear factor erythroid 2-related factor 2
NO	nitric oxide
Nox1	NADPH oxidase 1
Nox2	NADPH oxidase 2
Nox3	NADPH oxidase 3
Nox4	NADPH oxidase 4
Nox5	NADPH oxidase 5
PKC	protein kinase C
PN	peroxynitrite
Prdx1	peroxyredoxin1
PrdxIII	peroxyredoxin III
Prdx6	peroxyredoxin 6
siRNA	small interfering RNA
SOD	superoxide dismutase
TNFα	tumor necrosis factorα
NAC	N-acetylcysteine
NSC23766	N6-[2-[4-(Diethylamino)-1-methylbu-tyl]amino]-6-methyl-4-pyrimidinyl]-2-methyl- 4,6-qu-inolinediamine trihydrochloride
NOD	non-obese diabetic
Rac1	Ras-related C3 botulinum toxin substrate 1
RNS	reactive nitrogen species
ROS	reactive oxygen species
T1DM	type 1 diabetes mellitus
Tiam1	T-cell lymphoma invasion and metastasis-inducing protein 1
